# Environmental Health Literacy: an index to study its relations with pro-environmental behaviors

**DOI:** 10.1093/eurpub/ckac130.074

**Published:** 2022-10-25

**Authors:** A Carducci, M Fiore, C Lorini, I Federigi, M Verani, M Ferrante, G Bonaccorsi

**Affiliations:** 1 Department of Biology, University of Pisa, Pisa, Italy; 2 Department of Medical, Surgical Sciences and Advanced Technologies “G., University of Catania, Catania, Italy; 3 Department of Health Sciences, University of Florence, Florence, Italy

## Abstract

The citizen’ awareness about environmental health risks has been identified as an important determinant of citizens’ choices for the adoption of pro-environmental behaviors, but for its study simple measures to be applied in population studies are still lacking. The Environmental Health Literacy (EHL), is a recent sub-cathegory of health literacy, including functional, critical and interactive dimensions, that can be applied in surveys on environmental health risk perception and behaviors. The aim of our study was to elaborate and validate an EHL Index (ELHI) using data from a large multicenter survey carried out among 4778 students of different disciplines in 15 Italian Universities, with a self-administered anonymous questionnaire investigating risk perceptions, attitudes and behaviors towards environmental health risks and including a simple Functional Health Literacy test (FHL). From the original questionnaire of 56 items three sets of questions were selected to represent the three dimensions of health literacy (Functional, Critical or Interactive) and their outcomes were compared with the answers about FHL test and pro-environmental behaviors. The Principal Component Analysis was used to select the most representative questions that were then grouped in the EHLI. The index was significantly associated with both FHL test and behaviors questions. The ROC curve indicated a satisfying accuracy and was used to identify the best cut-off for ELHI. In conclusion the constructed ELHI can be considered reliable and useful for further population surveys in similar target people to plan communication interventions about environmental health risks and their prevention through individual choices.

**Key messages:**

- An Environmental Health Literacy Index is proposed as simple measure to be used in population surveys.
- This measure resulted associated with behaviors and can be considered accurate and reliable.

